# Mechanistic overview of protein-specific polysialylation

**DOI:** 10.1016/j.jbc.2025.110868

**Published:** 2025-10-28

**Authors:** Gaurang P. Bhide, Karen J. Colley

**Affiliations:** 1 Biologics CMC Development, AbbVie Bioresearch Center, Worcester, Massachusetts, USA; 2 Department of Biochemistry and Molecular Genetics, University of Illinois Chicago, Chicago, Illinois, USA

**Keywords:** glycoprotein biosynthesis, polysaccharide, sialic acid, sialyltransferase, glycosyltransferase

## Abstract

Polysialic acid is a polymer of α2,8-linked sialic acid residues attached to the termini of glycans that modify a select group of glycoproteins. It plays repulsive and attractive roles in nervous system and immune cell development and function, as well as cancer cell survival and metastasis and tissue regeneration, by negatively modulating the interactions of cells and proteins and by acting as a binding site to sequester biologically active molecules, respectively. An abundance of literature has shown that polysialylation is a protein-specific process in that the polysialyltransferases (polySTs) recognize and bind to sequences in one region of a protein substrate, which then allows them to polysialylate glycans in an adjacent region of that protein substrate. In this review, the requirements for the polysialylation of the neural cell adhesion molecule, synaptic cell adhesion molecule 1, and neuropilin 2 are discussed. The identification of acidic glycoprotein substrate sequences and corresponding basic polyST sequences that mediate the substrate–enzyme interaction is described, evidence for the direct interaction of these sequences in the case of the neural cell adhesion molecule is provided, conserved residues that may form a basic groove on the polyST surface that promotes polysialic acid chain polymerization are identified, and the role of polyST autopolysialylation in promoting glycoprotein substrate polysialylation is discussed. Finally, a perspective on how this information might be used to devise approaches to block or enhance polysialylation for therapeutic purposes is offered.

Polysialic acid (polySia) is a large carbohydrate polymer consisting of long chains of 8 to 400 α2, 8-sialic acid monomers that modify and terminate the α2,6- or α2,3-sialylated termini of N- and O-linked glycans found on a select set of mammalian glycoproteins (1, 2, 3, 4). Two transcriptionally regulated polysialyltransferases (polySTs) are responsible for the biosynthesis of polySia, ST8SiaII (also known as ST8Sia2 or STX) and ST8SiaIV (also known as ST8Sia4, PST, or PST-1) (5, 6). Unlike most glycosylation events, polysialylation is not simply dependent upon a glycosyltransferase recognizing a glycan acceptor on a protein as the protein traverses the secretory pathway. Instead, an initial protein–protein interaction between polyST and the glycoprotein substrate must occur before the polySia chain is initiated on the glycan acceptor attached to the protein. Consequently, polySia is found on a distinct group of glycoproteins, including the neural cell adhesion molecule (NCAM), the voltage-gated sodium channel α subunit, neuropilin 2 (NRP2), the synaptic cell adhesion molecule 1 (SynCAM1), the CD36 scavenger receptor in human milk, the C–C chemokine receptor-7 (CCR7), E-selectin ligand-1 (ESL-1), QSOX2, and the polySTs themselves (7, 8, 9, 10, 11, 12, 13, 14, 15, 16, 17).

While this review concerns the mechanism of mammalian polysialyation, it is important to note that polySia is also expressed by neuroinvasive bacteria as well as echinoderm and salmonid fish. PolySia is found on polysialoglycoproteins of Salmonidae fish eggs and on echinoderm (sea urchin) egg and sperm proteins, where it acts as a regulator of calcium concentration, thereby playing roles in sperm motility, the acrosome reaction, and embryogenesis (3, 18, 19, 20). The neuroinvasive bacteria, *Neisseria meningitides* serogroups B and C, express capsular α2,8- and α2,9-linked polySia, which allows these bacteria to evade the host immune response and thus is critical for their pathogenesis (2).

In mammals, polySia is expressed on cells of the nervous system, immune cells, cancer cells, and in various tissues during development, disease, and regeneration, where it plays both repulsive and attractive roles (2, 4, 21, 22, 23) (Fig. 1). Readers are encouraged to read other reviews in this issue that include discussions about the role of polySia in development, the nervous system, the immune system, cancer, and disease.

**Figure 1:**
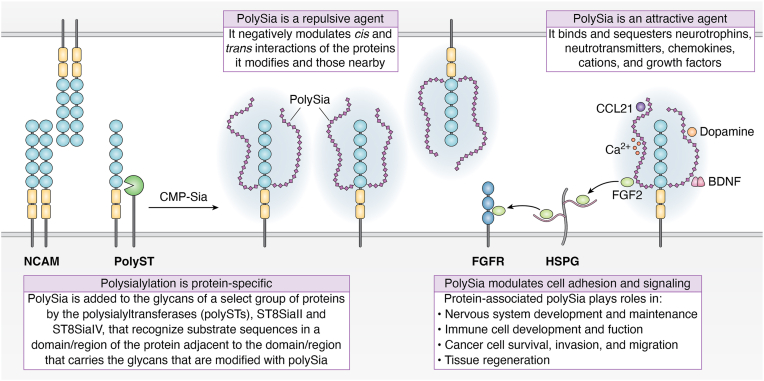
**PolySia biosynthesis and roles.** Polysialylation is a protein-specific modification that requires an initial protein–protein interaction between the polyST and the glycoprotein substrate before the glycans of the substrate are modified with polySia. The polySTs recognize specific residues in a domain/region of the glycoprotein substrate adjacent to the domain/region that carries the *N*- or *O*-linked glycans, which are terminated by polySia. Shown is the interaction between a polyST and NCAM with initial interaction of the polyST with the NCAM FN1 domain (*yellow rectangle*) and polymerization of the polySia chain (*purple diamonds*) on *N*-glycans in the adjacent Ig5 domain (*blue circle*). CMP–sialic acid (CMP–Sia) is the nucleotide sugar donor for the sialylation reaction. PolySia attached to glycoproteins serves as both a repulsive and attractive agent. It negatively modulates both *cis* and *trans* interactions of the proteins it modifies, like NCAM shown in the figure, as well as *trans* interactions of nearby proteins like E-cadherin and integrins (not shown). It is also an attractive agent that binds and sequesters biologically active molecules like neurotrophins (BDNF, NGF, NT3, and NT4), neurotransmitters (dopamine), growth factors (FGF2), chemokines (CCL21), and cations (Ca^2+^). The case of FGF2 binding to polySia is the most well understood. PolySia on NCAM binds FGF2 and transfers it to heparan sulfate on heparan sulfate glycoproteins (HSGPs), and this complex forms a ternary complex with the FGF receptor (FGFR), stimulating signaling. The ability of polySia to modulate protein–protein and cell–cell interactions and to sequester/deliver signaling molecules leads to its roles in a variety of essential biological events listed in the figure. For reviews, please see Refs. (2, 3, 4, 21, 23, 24, 27). CCL21, C–C motif ligand 21; FN, fibronectin; Ig, immunoglobulin-like domain; NCAM, neural cell adhesion molecule; polySia, polysialic acid; polyST, polysialyltransferase.

In this review, we report the in-depth analysis of the protein specificity of NCAM polysialylation and evaluate the requirements for the polysialylation of SynCAM1 and NRP2. Below we provide more details about NCAM, SynCAM1, and NRP2 and the role polySia plays in their function.

## Functional characteristics of polySia

The most highly expressed polysialylated protein is NCAM, and the role of polySia in NCAM function has been extensively studied. Based on these studies, polySia has been called a “global regulator of cell adhesion” (24). PolySia is capable of binding large amounts of water, leading to an increase in the hydrodynamic radius of the polysialylated protein (24, 25). Indeed, it functions as a repulsive agent that blocks the cell adhesion events mediated by these proteins (like NCAM), as well as other adhesive interactions of nearby proteins, such as E-cadherin, on the same cell surface (26) (Fig. 1). PolySia is also a modulator of select signaling pathways because it is able to regulate homophilic and heterophilic protein–protein interactions between cells and on the same cell surface, and because it can serve as an attractive agent able to bind and sequester biologically active molecules like neurotrophins, neurotransmitters, cytokines, and growth factors, driven by its negative charge (reviewed in Refs. (2, 4, 27)) (Fig. 1).

### The biological roles of polySia on NCAM

NCAM polysialylation is developmentally regulated, and polysialylated NCAM plays a role in brain formation and nervous system maintenance (2, 4, 21), immune cell development and function (22), and cancer cell survival, migration, and invasion (23, 28) (Fig. 1). As an illustration of one role polysialylated NCAM plays, we will briefly discuss the dynamics and function of polysialylated NCAM in the brain and nervous system. NCAM is highly polysialylated in the embryonic and perinatal brain and is expressed by neural precursors and immature neurons (29, 30). Polysialylation is dramatically decreased in the postnatal brain but is found in locations where ongoing neurogenesis or plasticity is occurring, such as the hippocampus, hypothalamus, and olfactory bulb (2, 4, 31, 32, 33, 34, 35).

Analysis of mice lacking the two polySTs and/or NCAM demonstrated that polySia plays roles in axon guidance and neural cell migration by modulating NCAM interactions during development (36). Cremer *et al.* (37, 38) showed that mice lacking NCAM expression have mild phenotypes, including impaired postnatal migration of interneuron precursors from the subventricular zone toward the olfactory bulb, the incorrect lamination of hippocampal mossy fibers, and deficits in spatial learning. These defects were also seen when an endosialidase that specifically cleaves polySia was injected into the postnatal brain (39, 40). In contrast, elimination of both polySTs in mice results in smaller overall body and brain size, a reduction in olfactory bulb size, defects in motor neuron fasciculation and axon guidance, hydrocephaly, and death within 4 weeks of birth. Knocking out NCAM in the double polyST knockout reversed these defects, suggesting that the loss of polySia led to inappropriate NCAM interactions that altered axon guidance and neural cell migration and subsequent nervous system development (36). More recently, it has been appreciated that altered polySia–NCAM levels lead to changes during development of the nervous system and a predisposition to various psychiatric diseases, and that polySia–NCAM levels in the brain impact learning and memory, aging, and neurodegeneration (21, 41).

### The biological roles of polySia on other substrates

SynCAM1 is an immunoglobulin superfamily protein that mediates calcium-dependent interactions critical for excitatory synapse formation and synapse plasticity (42, 43). Galuska *et al.* (11) found that a small proportion of SynCAM1 in mouse brain is polysialylated and that this polySia–SynCAM1 is exclusively expressed by a population of NG2 glial cells, which are oligodendrocyte precursor cells that are the source of myelinating oligodendrocytes in the central nervous system (11, 21). SynCAM1 is comprised of three immunoglobulin-like (Ig) domains, a transmembrane region, and a short cytoplasmic tail. PolySia is found on N-glycans in the first Ig domain of SynCAM1, and its presence was shown to block homophilic binding *in vitro* (11). Rollenhagen *et al.* (44) later showed that SynCAM1 is exclusively polysialylated by ST8SiaII in mice, although ST8SiaIV can weakly polysialylate SynCAM1 *in vitro*.

The neuropilins, NRP1 and NRP2, are coreceptors for vascular endothelial growth factor (VEGF) and class 3 semaphorins, as well as other growth factors, and serve to modulate their interactions with receptors (45, 46). NRP1 is essential for vascular development and angiogenesis, whereas NRP2 plays roles in the development of the lymphatic system and in axon guidance (46). Curreli *et al.* (10) showed that NRP2 is polysialylated on O-glycans by ST8SiaIV in dendritic cells, which are antigen-presenting cells that migrate to the lymph node to activate T cells (47). Their work suggested that the presence of polySia on NRP2 blocked T-cell activation and proliferation. However, later work by two groups (48, 49) suggested that polysialylated NRP2 binds to the C–C motif ligand 21, presenting this chemokine to its receptor CCR7 on mature dendritic cells. This in turn stimulates dendritic cell migration to the lymph node and T-cell activation. Kiermaier *et al.* (14) then demonstrated that the CCR7 chemokine receptor is itself polysialylated on N- and O-glycans by ST8SiaIV in dendritic cells. Their studies suggested that the polySia on CCR7 stimulates the release of autoinhibition of the chemokine C–C motif ligand 21, allowing it to bind to CCR7, promoting dendritic cell trafficking to the lymph node (22).

Some cells express multiple polysialylated proteins simultaneously. For example, Werneberg *et al.* (13, 50) demonstrated that NRP2 and ESL-1 glycans are modified with polySia in cultured microglia and THP-1 macrophages and in injury-induced microglia in brain slice cultures. They showed that polysialylated ESL-1 and NRP2 accumulate in the Golgi of cultured microglial and THP-1 macrophages and are rapidly translocated to the plasma membrane following lipopolysaccharide stimulation, where they are cleaved and shed into the extracellular space. Other studies by Thiesler *et al.* (51) indicate that the interaction of these cleaved polysialylated proteins with murine Siglec-E leads to the inhibition of activated microglia.

### Similarities with other protein-specific glycosylation events

Abundant evidence has demonstrated that polysialylation is a protein-specific modification that is initiated by a protein–protein interaction between polyST and a glycoprotein substrate. There are other notable examples of protein-specific glycan modifications. The biosynthesis of the mannose-6-phosphate recognition marker required for lysosomal enzyme trafficking to the lysosome is a two-step process that is initiated by the *N*-acetylglucosmine-1-phosphotransferase recognizing a signal patch comprised of specifically spaced lysine and arginine residues on the lysosomal enzyme (52, 53, 54). The generation of a terminal GalNAc-4-SO_4_ structure on the *N*-glycans of the α-subunit of pituitary glycoprotein hormones, a modification required for the clearance of these enzymes from the circulation, requires that an *N*-acetyl galactosaminyltransferase recognizes an α-helical stretch of basic residues six to nine amino acids N terminal to the *N*-glycan that is modified (55, 56, 57). Two other modifications depend on structural elements being recognized by modifying enzymes—the addition of a terminal glucose and the generation of Glc_1_Man_9_GlcNAc_2_ oligosaccharides on misfolded/unfolded glycoproteins in the endoplasmic reticulum that allows their interactions with the chaperones calnexin and calreticulin (58, 59), and the addition of O-fucose to epidermal growth factor repeats of proteins such as Notch (60, 61). Like these protein-specific glycan modification events, polysialylation requires the polySTs to recognize protein substrate sequences before modifying the protein’s glycans.

### The polySTs and their relationship to other α2,8-sialyltransferases and each other

To date, six α2,8-sialyltransferases have been cloned. ST8SiaI and ST8SiaV have been shown to exclusively modify gangliosides (62, 63, 64, 65, 66). The polySTs, ST8SiaII and ST8SiaIV, which share 59% sequence identity, were cloned by multiple groups in the mid-1990s and shown to polysialylate NCAM glycans (reviewed in Refs. (5, 6)). In 2000, Angata *et al.* (67) cloned ST8SiaIII and showed that it could add shorter chains of sialic acid (oligosialic acid, less than eight sialic acid units) to the glycans of NCAM and some other non-NCAM acceptors. In addition, these investigators demonstrated that ST8SiaIII was able to autocatalytically polysialylate its own glycans but did not polysialylate NCAM (67). In 2005, Teintenier-Lelievre *et al.* (68) cloned the remaining enzyme, ST8SiaVI, and demonstrated that it synthesizes a di-sialic acid structure on *O*-glycans.

The two polySTs are both independently able to polysialylate NCAM but are believed to work together during embryogenesis when NCAM is most highly polysialylated (2, 3, 4, 5, 6). However, their levels vary during this time, and studies point to ST8SiaII dominating in the developing brain. The polySTs are also expressed in different locations in the adult brain and also differ in their cell- and tissue-specific expression during postnatal development and in fetal and adult extraneural tissues with some overlap (reviewed in Refs. (2, 3, 4, 5, 6)). While ST8SiaIV has been found to synthesize longer polySia chains on NCAM glycans *in vitro* (69), both polySTs are able to synthesize chains of 50 sialic acid units or greater on NCAM glycans *in vivo* (70). In addition, both *in vitro* and *in vivo* studies suggest that the polySTs may coordinately act to form longer polySia chains on NCAM (69, 70, 71).

One of the goals of this review is to help the readers appreciate the complexity and uniqueness of polysialylation machinery. First, like other glycosylation events, polysialylation is not template driven and demands stereospecificity. Second, given its protein-specific nature, the polySTs have the task of recognizing and binding the select proteins they identify as substrates in the face of a vast pool of plasma membrane and secreted proteins that also pass through the secretory pathway. Third, because polySia is a polymer, once polySTs have recognized and bound their substrates, they synthesize the polySia chain monomer by monomer using the CMP–sialic acid donor. As a result, the substrate–polyST interaction should be strong enough to hold on to the substrates while polySia chains are being synthesized but weak enough to release the completely modified substrate. In the upcoming sections, we will review biochemical, biophysical, and structural studies that have been done to characterize the process of protein-specific polysialylation and discuss how these findings could be used for therapeutic purposes.

## Protein-specific polysialylation: protein sequences required for polyST recognition and polysialylation

In 2003, the Colley laboratory set out to test the hypothesis that polysialylation is a protein-specific modification that requires the polySTs to recognize and bind to a protein substrate before modifying its glycans. The possibility that the polySTs recognize their protein substrates before modifying substrate glycans was suggested by early work. In 1995, Kojima *et al.* (72) first showed that NCAM was a far better substrate for ST8SiaII compared with fetuin or other proteins that carried α2,3- and α2,6-sialylated *N*-glycans. Further characterization revealed that *N*-glycans isolated from NCAM were not substrates for ST8SiaII or ST8SiaIV, suggesting these enzymes may recognize the NCAM protein and not just the glycan acceptor on NCAM (67, 73, 74). For example, Angata *et al.* (67) evaluated the substrate specificity of ST8SiaIV and ST8SiaII and found that both enzymes could add sialic acid to free oligosaccharides *in vitro*, but that the efficiency increased 1500-fold when they added sialic acid to oligosaccharides on the NCAM protein. In addition, Close *et al.* (75) demonstrated that when a catalytically active but nonautopolysialylated ST8SiaIV was expressed in COS-1 cells, no proteins in these cells were polysialylated; only when NCAM was coexpressed with this nonautopolysialylated ST8SiaIV mutant were polysialylated proteins (polySia–NCAM) detected in the cells. Taken together, these data strongly suggested that polysialylation is a protein-specific modification event. Like other protein-specific modification events, it was expected that specific sequences in the glycoprotein substrate would be recognized by the polySTs. Subsequent work has demonstrated that this is indeed the case.

### NCAM domains required for polysialylation

There are three isoforms of NCAM, all with the same extracellular structure that consists of five Ig domains and two fibronectin (FN) type III repeats (76). NCAM180 and NCAM140 have a transmembrane region and cytoplasmic tails of differing length, whereas NCAM120 is a glycosylphosphatidylinositol-anchored protein. NCAM140 was used in the studies described below. Work by Nelson *et al.* (77) demonstrated that NCAM has six sites of *N*-linked glycosylation, and sites 5 and 6 (Asn^449^, Asn^478^) on Ig5 carry the majority of the polySia.

To determine which NCAM domains were essential for polysialylation, the ability of the two polySTs to polysialylate a series of NCAM domain deletion mutants was evaluated. In 1995, before the polyST complementary DNAs were cloned, Nelson *et al.* (77) expressed NCAM mutants in F11 rat/mouse hybrid cells capable of polysialylating exogenous, full-length NCAM, and concluded that the minimum NCAM extracellular domain structure for polysialylation was Ig4–Ig5–FN1 and that NCAM membrane association was also essential for efficient polysialylation. Later work by this group suggested that Ig4 is not necessary for polysialylation of the *N*-glycans on Ig5 (78). In 2002, using recombinant enzymes, Angata *et al.* (69) again suggested that Ig4 was necessary for polysialylation by ST8SiaIV and that ST8SiaII only weakly polysialylated an NCAM Ig4–Ig5–FN1–FN2 domain deletion mutant.

**Figure 2:**
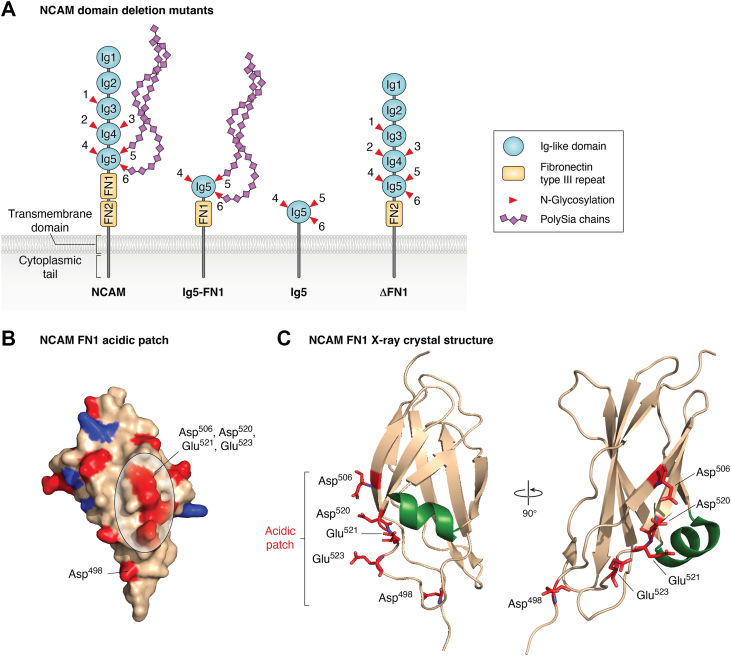
**Requirements for NCAM polysialylation: an acidic patch on the first fibronectin type III repeat (FN1) is required for the polysialylation of *N*-glycans on the adjacent Ig5 domain.***A*, NCAM domain deletion mutants. NCAM consists of five Ig-like domains (Ig1–5), two fibronectin type III repeats (FN1 and FN2), a transmembrane domain, and a cytoplasmic tail. There are six sites of *N*-glycosylation indicated by the *inverted triangles*, and the glycans on Asn5 and Asn6 carry the bulk of the polySia. Domain deletion mutants that include Ig5 and FN1 are polysialylated like full-length NCAM. Proteins with Ig5 but lacking FN1 are not polysialylated, indicating that the FN1 domain is essential for the polysialylation of the glycans on Asn5 and Asn6 in the Ig5 domain (79). PolySia chains are indicated by *purple diamonds*. *B*, an acidic patch unique to NCAM FN1 is in *red* with Asp^498^ indicated and Asp^506^, Asp^520^, Glu^521^, and Glu^523^*circled* (86). *C*, two views of the structure of the NCAM FN1 domain highlighting the unique α-helix in *green* and the acidic patch comprised of Asp^498^, Glu^506^, Asp^520^, Glu^521^, and Glu^523^ in *red*. Note that Asp^520^, Glu^521^, and Glu^523^ were the residues first identified in the acidic patch, and the other two residues were identified later. The unique FN1 α-helix is shown in *green*. FN, fibronectin; Ig, immunoglobulin-like domain; NCAM, neural cell adhesion molecule; polySia, polysialic acid.

To get a sense of which domains might contain the interaction sites for the polySTs, Close *et al.* (79) evaluated requirements for NCAM polysialylation by ST8SiaIV and ST8SiaII using a series of soluble and membrane-associated NCAM domain deletion mutants (see Fig. 2*A* for select mutants). An NCAM protein consisting of only Ig5–FN1 was polysialylated by both polySTs to levels equivalent to those of an Ig4–Ig5–FN1 protein, whereas Ig5 alone (NCAM5) was not polysialylated by either enzyme, suggesting that the FN1 domain is essential for NCAM polysialylation (Fig. 2*A*). Soluble forms of both Ig4–Ig5–FN1 and Ig5–FN1 were polysialylated, but to a much lower extent, resulting in proteins that migrated with smaller molecular masses than the equivalent membrane-associated proteins. This suggested that membrane association was not absolutely required for polysialylation, but it did enhance the amount of sialic acid added. Two other interesting observations were made—that a protein consisting of just the five NCAM Ig domains was weakly polysialylated on *N*-glycans by ST8SiaIV, but not by ST8SiaII, and that a protein consisting of just the two NCAM FN domains was weakly polysialylated on O-glycans by ST8SiaIV but not by ST8SiaII. At the time, the significance of these observations was not clear, but as you will see, they helped explain later data.

### NCAM FN1 domain requirements for polysialylation

FN domains are found in 2% of all proteins and are regions of structural and not sequence homology (80, 81, 82). Of all the proteins that contain FN domains only, NCAM is polysialylated, suggesting that specific sequences rather than structural elements are required for polyST recognition. To understand which sequences in NCAM FN1 play a role in polyST recognition and binding, multiple approaches were taken that involved comparing the structures and sequences of FN domains that did and did not support polysialylation. First, comparison of NCAM’s FN1 and FN2 domains demonstrated structural similarity but not sequence similarity. A mutant protein in which the FN1 domain of NCAM was deleted (Fig. 2*A*, ΔFN1), effectively moving FN2 adjacent to Ig5, was not polysialylated by either polyST. In addition, the ΔFN1 protein did not bind ST8SiaIV, whereas full-length NCAM did bind ST8SiaIV (83, 84). These experiments demonstrated that the NCAM FN2 domain could not replace the NCAM FN1 domain to support polyST recognition/binding and the polysialylation of NCAM.

Modeling the NCAM FN1 structure on the known crystal structure of the rat NCAM FN2 domain revealed a unique acidic surface patch in FN1 not found in FN2 (Fig. 2*B*) (85). Replacing three acidic residues (Asp^520^, Glu^521^, and Glu^523^) with arginine residues eliminated the polysialylation of the Ig5–FN1 tandem and full-length NCAM. However, replacing these residues with alanine residues eliminated the polysialylation of the Ig5–FN1 tandem but not the full-length protein, suggesting that additional residues might play a role in polyST recognition of the FN1 domain. An additional possibility was that the membrane-associated Ig5–FN1 tandem that is lacking the FN2 domain was not optimally positioned vis-á-vis the membrane-associated polyST, leading to a strain on recognition. In support of this notion and highlighting the importance of the positioning of the membrane-associated polyST and substrate, replacing the FN1 acidic residues with alanine residues in the Ig5–FN1–FN2 protein did not significantly impact its polysialylation (83).

Mendiratta *et al.* (86) solved the crystal structure of human NCAM FN1 in order to determine what the larger FN1 recognition region might include. This structure validated the presence of an acidic patch that included Asp^520^, Glu^521^, and Glu^523^ as well as Asp^498^ (Asp^506^ was later included in the acidic patch) and revealed unique α-helix linking strands 4 and 5 of its β-sandwich structure (Fig. 2*C*). Replacing the α-helix with threonine or alanine residues did not eliminate polysialylation but shifted polySia addition from the *N*-glycans on Ig5 to *O*-glycans on FN1. Replacing acidic patch residues had the same impact on polysialylation of *N*-glycans in full-length NCAM and *O*-glycans in the Δhelix mutants (86). These data suggested that the α-helix, while not part of a recognition sequence, may be required to position the polyST in a way that allows it to polysialylate the Ig5 *N*-glycans.

**Figure 3:**
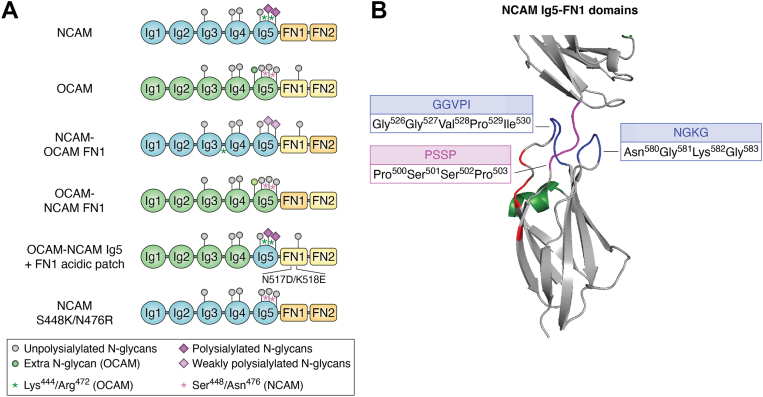
**NCAM–OCAM chimeras: polyST interactions with both NCAM FN1 and Ig5 domains are essential for polysialylation.** OCAM is highly homologous to NCAM and expressed at some of the same times and locations as NCAM but is not polysialylated. *A*, a series of chimeric and mutant proteins were created, coexpressed with ST8SiaIV, and evaluated for polysialylation (92). An NCAM–OCAM FN1 chimera was polysialylated at approximately 50% the level of wildtype NCAM. However, an OCAM–NCAM FN1 chimera was not polysialylated, suggesting that sequences outside the FN1 domain may be essential for, or must be permissive for, recognition and modification. OCAM contains an extra *N*-glycan in the Ig5 domain (*green ball*) and two highly charged residues adjacent to or nearby Asn6 and Asn7, the glycosylation sites analogous to those in NCAM that are polysialylated (Asn5 and Asn6). *Pink stars* represent Lys^444^ near Asn6 (Asn^445^) and Arg^472^ near Asn7 (Asn^474^) in OCAM, whereas *green stars* represent Ser^448^ near Asn5 (Asn^449^) and Asn^476^ near Asn6 (Asn^478^) in NCAM. Placing these highly charged residues in NCAM Ig5 in the S448K–N476R mutant eliminates NCAM polysialylation and decreases binding to ST8SiaIV. While elimination of the “extra” glycosylation site in OCAM FN1 does not promote polysialylation, and replacing the OCAM Ig5 with NCAM Ig5 in the OCAM–NCAM Ig chimera allows only weak polysialylation (data not shown), recreating the FN1 acidic patch with the replacement of Asn^517^ with Asp and Lys^518^ with Glu (N517D/K518E) in the OCAM–NCAM Ig5 chimera allows robust polysialylation (OCAM–NCAM Ig5 + FN1 acidic patch). *Gray balls* on *black sticks* represent unpolysialylated *N*-glycans; *purple balls* on *black sticks* represent polysialylated *N*-glycans; *light purple balls* on *black sticks* represent weakly polysialylated *N*-glycans. *B*, residues common to both NCAM and OCAM shown on the structure of NCAM Ig5–FN1 include GGVPI and NGKG loops (in *blue*) that are adjacent to PSSP (in *magenta*) that is part of the Ig5–FN1 linker region. The unique FN1 α-helix is shown in *green*, and the FN1 acidic patch residues are shown in *red*. PSSP sequences play a role directly or indirectly in polyST recognition/binding, whereas the GGVPI and NGKG sequences form loops that stabilize the Ig5–FN1 linker. Replacing each region with stretches of alanine residues eliminates NCAM polysialylation, but only the PSSP alanine replacement decreases polyST binding. FN, fibronectin; GGVPI, Gly-Gly-Val-Pro-Ile; Ig, immunoglobulin-like domain; NCAM, neural cell adhesion molecule; NGKG, Asn-Gly-Lys-Gly; OCAM, olfactory cell adhesion molecule; PolyST, polysialyltransferase; PSSP, Pro-Ser-Ser-Pro.

A chimeric protein approach using sequences from an unpolysialylated adhesion molecule, the olfactory cell adhesion molecule (OCAM), provided key information about how the polySTs recognize NCAM (87). OCAM is an adhesion protein expressed on neurons of the olfactory system and by other tissues like the retina (88, 89). OCAM, also known as Rb-8 neural cell adhesion molecule, NCAM2 (90), and mammalian fasciulin II (91), has an identical extracellular domain structure as NCAM and even *N*-glycosylation sites that match Asn5 and Asn6 in its Ig5 domain, but it is not polysialylated (88, 89, 90) (Fig. 3*A*). OCAM FN1 shares 37% sequence identity with NCAM FN1 (88) but lacks the NCAM FN1 α-helix and acidic patch.

Unexpectedly, Foley *et al.* (87) found that an NCAM–OCAM FN1 chimera, where OCAM FN1 replaced NCAM FN1, was polysialylated, but only at about 50% of that observed with full-length NCAM (87) (Fig. 3*A*, NCAM–OCAM FN1), suggesting that at least some portion of the recognition site was present in OCAM FN1. Thompson *et al.* (84) found that NCAM FN1 and OCAM FN1 had three stretches of amino acids in common: Pro-Ser-Ser-Pro (PSSP), Gly-Gly-Val-Pro-Ile (GGVPI), and Asn-Gly-Lys-Gly (NGKG) (Fig. 3*B*). The PSSP sequence is adjacent to the Ig5–FN1 linker region, whereas GGVPI and NGKG form loops on either side of the Ig5–FN1 linker. Replacing any of these three stretches with alanine residues dramatically reduced NCAM polysialylation. Replacing PSSP or the acidic patch residues decreased NCAM–ST8SiaIV binding. While replacing either the GGVPI or NGKG residues did not impact NCAM–polyST binding (84). These results suggested that while PSSP could be part of a polyST recognition region, the GGVPI and NGKG loops might serve to stabilize the Ig5–FN1 linker region (Fig. 3*B*).

Further inspection of the OCAM FN1 domain revealed that two of the three core acidic patch residues found in NCAM FN1 were missing. NCAM Asp^520^ was replaced by Asn^517^ in OCAM, and NCAM Glu^521^ was replaced by Lys^518^ in OCAM (92). Recreating the acidic patch in the OCAM FN1 domain in the OCAM–NCAM Ig5 chimera that was only weakly polysialylated increased the chimera’s polysialylation by 66% and highlighted the importance of the FN1 acidic patch in polyST recognition and NCAM polysialylation (92) (Fig. 3*A*. OCAM–NCAM Ig5 + FN1 acidic patch).

### The importance of the Ig5–FN1 relationship for NCAM polysialylation

**Figure 4:**
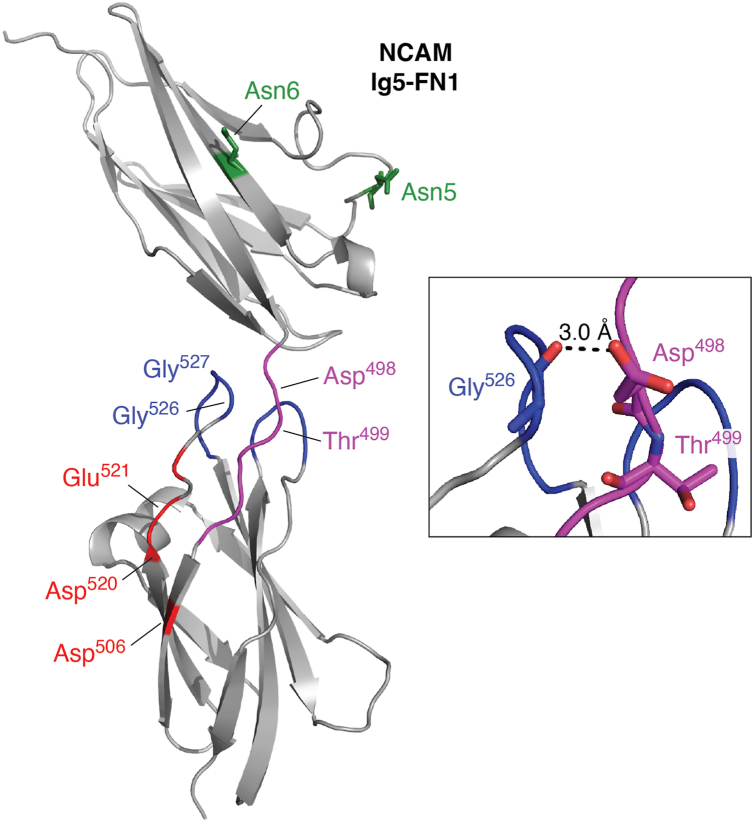
**NCAM Ig5–FN1 structure and stabilizing interactions between the GGVPI loop and the Ig5–FN1 linker region.** The NCAM Ig5–FN1 structure is shown (Protein Data Bank entry: 3MTR) (see Ref. (93) for details). Asn residues modified by glycans that are polysialylated are shown in *green* (Asn5, Asn6). Ig–FN1 linker, including the PSSP sequence and Asp^498^, is colored *magenta*, the GGVPI and NGKG loops flanking the linker are colored *blue*, acidic patch residues Glu^506^, Asp^520^, Glu^521^, and Glu^523^ (*unlabeled*) are shown in *red*. Residues perturbed by interactions between the NCAM FN1 domain and the ST8SiaIV PBR are labeled (Asp^498^, Thr^499^, Asp^506^, Asp^520^, Glu^521^, Gly^526^, and Gly^527^). *Inset,* the region between Ig5–FN1 linker and GGVPI loop is enlarged to show the side chain of Asp^498^ forming a hydrogen bond with the main chain carbonyl of Gly^526^ (87). FN, fibronectin; GGVPI, Gly-Gly-Val-Pro-Ile; Ig, immunoglobulin-like domain; NCAM, neural cell adhesion molecule; NGKG, Asn-Gly-Lys-Gly; PBR, polybasic region.

The positioning of the Ig5 and FN1 domains seems critical for the NCAM polysialylation process because inserting just three amino acids in the linker between these two domains eliminated the polysialylation of the NCAM–OCAM FN1 chimera (87). The crystal structure of the NCAM Ig5–FN1 tandem provided no evidence that the α-helix mediated interactions between the Ig5 and FN1 domains, making it more likely that the α-helix enabled appropriate interactions between the NCAM FN1 domain and the membrane-bound polyST for the polysialylation of Ig5 *N*-glycans (93). In addition, the structure showed that the Asn5 and Asn6 that carry the polysialylated *N*-glycans are located on the opposite face from the acidic patch and the α-helix of FN1, whereas Asn4, which carries an *N*-glycan that is very weakly polysialylated, is found to more closely align with the FN1 acidic patch and α-helix (Fig. 4).

NCAM proteins lacking Asn5 and Asn6 but containing new *N*-glycosylation sites spread over the Ig5 surface were all polysialylated, but never to the extent of wildtype NCAM, and the placement of these sites and the extent of their polysialylation did not depend on their proximity/alignment with Asn5 or Asn6 or distance from the membrane, suggesting that the polySTs can engage with several sites on FN1 and/or the orientation of the Ig5 and FN1 domains is flexible (93). Indeed, evidence had been provided to suggest that the NCAM extracellular domain contained a flexible hinge. Rotary shadowing experiments by Becker *et al.* (94) suggested that there is a flexible hinge in NCAM’s extracellular portion nearby sites of polysialylation (94, 95). Later, direct force measurements by Johnson *et al.* (96) were most consistent with the flexible hinge being between Ig5 and FN1 (94), and still others believed that the flexible hinge was between Ig4 and Ig5 (97, 98). Finally, a structure of FN1–FN2 showed a great degree of flexibility between these domains and suggested that the flexible hinge might be between these two domains (99).

### Role of Ig5 sequences in polyST recognition and polysialylation

The finding that the OCAM FN1 domain could partially replace the NCAM FN1 domain to support recognition and polysialylation of the chimera begged the question of why OCAM was not polysialylated and suggested that the OCAM Ig5 domain is not permissive for polysialylation (88). This idea was supported by the observation that the OCAM–NCAM FN1 chimera, where NCAM FN1 replaced OCAM FN1, was not polysialylated, whereas an OCAM–NCAM chimera where NCAM Ig5 replaced OCAM Ig5 was weakly polysialylated (92) (Fig. 3*A*, OCAM–NCAM FN1 and OCAM–NCAM Ig5). Along these lines, two glycosylation mutants analyzed in the aforementioned study of Foley *et al.* (93) were polysialylated, but not on *N*-glycans but on *O*-glycans, likely in the FN1 domain. Could the presence of these *N*-glycans have altered polyST interactions with the Ig5 domain that are important for polysialylation of Ig5 *N*-glycans? Comparison of the NCAM and OCAM Ig5 domains showed that OCAM Ig5 has an “extra” *N*-glycan and that there are two large basic residues located near OCAM Ig5 Asn6 and Asn7 (analogous to NCAM Asn5 and Asn6). Thompson *et al.* (92) found that adding the extra *N*-glycan attachment site to NCAM Ig5 blocked NCAM polysialylation, but removing this *N*-glycosylation site from OCAM Ig5 was not sufficient to recover OCAM polysialylation.

Ultimately it was discovered that the two large basic residues found adjacent or nearby to OCAM Ig5 Asn6 and Asn7 sites of N-glycosylation were blocking OCAM polysialylation. A serine residue (Ser^448^) found adjacent to Asn5 (Asn^449^) in NCAM Ig5 is replaced by a lysine residue (Lys^444^) adjacent to Asn6 (Asn^445^) in OCAM Ig5. Similarly, an asparagine residue (Asn^476^) two residues away from Asn6 (Asn^478^) in NCAM Ig5 is replaced by an arginine residue (Arg^472^) that is two residues away from Asn7 (Asn^474^) in OCAM Ig5. Inserting these large basic residues into the NCAM Ig5 domain alone or together substantially reduced or nearly eliminated NCAM polysialylation and substantially reduced ST8SiaIV–NCAM interactions as measured by coimmunoprecipitation (92) (Fig. 3*A*, NCAM S448K/N476R). Taken together, these results indicate that while the polySTs require an interaction with the acidic patch in the NCAM FN1 domain, secondary interactions with residues in the Ig5 domain are necessary for the polysialylation of *N*-glycans in this domain.

## Protein-specific polysialylation: PolyST sequences required for substrate recognition

The polySTs are unique compared with other α2,8-sialyltransferases in that they have three specific roles: (i) substrate protein recognition, (ii) polySia chain initiation, and (iii) polySia chain elongation. Of all the α2,8-sialyltransferases, ST8SiaIII is most closely related to ST8SiaII and ST8SiaIV (35% sequence identity with ST8SiaIV). Evaluation of NCAM polysialylation by ST8SiaIII/IV chimeric enzymes allowed Angata *et al.* (100) to identify an ST8SiaIV N-terminal region comprised of amino acids 62 to 127 and a region close to the catalytic domain, comprised of residues 194 to 267, both unique to polySTs, that were important for NCAM polysialylation. Given the location of disulfide bonds in ST8SiaIV, these regions were believed to be in proximity to one another in the folded protein (see the model in the study by Angata *et al.* (5)).

### Role of specific basic residues in polysialyltransferase domain and polybasic region regions in NCAM polysialylation

**Figure 5:**
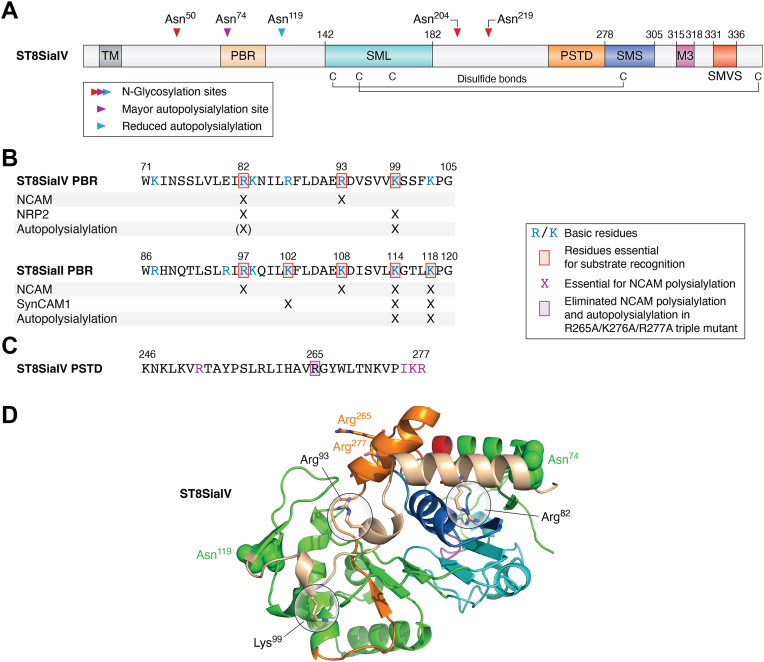
**PolyST conserved sequences, autopolysialylation, disulfide bonds, key sequences, and modeled structure.***A*, the polySTs, ST8SiaII and ST8SiaIV, are type II, Golgi membrane proteins with short amino-terminal cytoplasmic tails followed by transmembrane (TM) regions, stem regions that are susceptible to cleavage, and catalytic regions that include stretches of amino acids conserved in other sialyltransferases (sialyl motifs) and sequences exclusively found in polySTs (polybasic region [PBR] and polysialyltransferase domain [PSTD]). Shown is a schematic for ST8SiaIV. Sialyl motifs include the sialyl-motif large (SML) in *cyan*, including amino acids 142 to 182; sialyl-motif small (SMS) in *marine*, including amino acids 278 to 305; motif III (M3) in *magenta*, including amino acids 315 to 318, and sialyl-motif very small (SMVS) in *red*, including amino acids 331 to 336. His^331^ in SMVS is essential for polyST activity. ST8SiaIV has five sites of *N*-glycosylation (Asn^50^, Asn^74^, Asn^119^, Asn^204^, and Asn^219^), with the glycan on Asn^74^ (*red*) serving as the major site of autopolysialylation and the glycan on Asn^119^ showing significantly less autopolysialylation (*cyan*). ST8SiaIV also has five Cys residues, four of which form disulfide bonds (Cys^142^–Cys^292^ and Cys^156^–Cys^356^) that are believed to bring the sialyl motifs that are required for catalytic activity and glycan substrate binding together (100) (see model in Ref. (100)). *B*, the PBR sequences of ST8SiaII and ST8SiaIV contain basic residues essential for substrate recognition. Basic residues are in *blue*, those residues essential for substrate recognition are *boxed*, and their contributions to substrate polysialylation and polyST autopolysialylation are indicated by X, with (X) indicating a lesser contribution. Please see text for a detailed summary. *C*, the polysialyltransferase domain (PSTD) of ST8SiaIV. This region extends from Lys^246^ to Arg^277^ and is immediately adjacent to the SMS. Residues identified by Nakata *et al.* (109) as essential for NCAM polysialylation are shown in *violet*, and an additional residue (Arg^265^, *boxed*) is shown that was replaced by Foley *et al.* (110) in a triple mutant R265A/K276A/R277A that eliminated NCAM polysialylation and autopolysialylation. *D*, a model of ST8SiaIV based on the X-ray crystal structure of ST8SiaIII (Protein Data Bank entry: 5BO6), generated using SWISS-MODEL (118, 141, 142, 143, 144, 145). The PBR region is depicted in *wheat*, the PSTD region is depicted in *orange*. Sialyl motifs are depicted in *cyan* (SML), *marine* (SMS), *red* (SMVS), and *violet* (motif III). Asn attachment sites of the autopolysialylated *N*-glycans (Asn^74^ and Asn^119^) are shown as *green spheres*. Key residues in the PBR (Arg^82^, Arg^93^, and Lys^99^) are labeled in *black*, Arg^265^ and Arg^277^ from the PSTD are labeled in *orange*. NCAM, neural cell adhesion molecule; polyST, polysialyltransferase.

The polySTs, like all Golgi glycosyltransferases, are type II membrane proteins with amino-terminal cytoplasmic tails, transmembrane regions, and carboxy-terminal sequences facing the Golgi interior that include an extended stem region followed by a large catalytic domain (101) (Fig. 5*A*). Sialyltransferases, including the polySTs, include three conserved motifs required for catalytic activity (102, 103, 104). These include the sialyl-motif large that is predicted to bind the CMP-Sia donor (105), the sialyl-motif small (SMS) that is believed to bind both the donor and glycan acceptor (106), and the sialyl-motif very small that contains a conserved histidine residue that is required for catalytic activity (104, 107). Conserved histidine and tyrosine residues are found in a fourth conserved sequence of four amino acids called motif III that has also been suggested to be required for glycan acceptor recognition and enzyme activity (108) (Fig. 5*A*).

Nakata *et al.* (109) found that a 32 amino-acid region upstream of SMS exhibited a particularly high pI (∼12) because of the abundance of basic residues in this region. They named it the polysialyltransferase domain (PSTD), given its unique existence in the polySTs but not in ST8SiaIII or other α2,8-sialyltransferases (Fig. 5, *A* and *C*, ST8SiaIV PSTD). They reasoned that given its proximity to the SMS, this region must be important for the processive synthesis of a negatively charged polySia polymer. Their studies showed that mutating Lys^250^, Arg^252^, His^265^, Lys^272^, Ile^275^, Lys^276^, and Arg^277^ in the PSTD variably influenced polysialylation of NCAM-Fc in an *in vitro* assay. However, given the proximity of PSTD residues to CMP–sialic acid donor and glycan acceptor–binding regions of polySTs, it was unclear whether PSTD residues also played a role in polyST catalytic activity (109).

Searching for a basic polyST region that could bind the FN1 acidic patch of NCAM, Foley *et al.* (110) identified a stretch of basic residues conserved in both polySTs that extended from Trp^71^–Gly^105^ in ST8SiaIV and Trp^86^–Gly^120^ in ST8SiaII and called this region the “polybasic region” or PBR (Fig. 5*B*). Like the PSTD, the PBR is unique to the polySTs and contains seven to eight basic residues within the span of 35 residues. Of the seven basic residues in ST8SiaIV PBR, replacing Arg^82^ and Arg^93^ (R82A/R93A) significantly reduced NCAM polysialylation. Replacing these basic residues with another basic residue, lysine, in the R82K/R93K mutant maintained NCAM polysialylation, suggesting the importance of the positive charge in these positions. In addition, the R82A/R93A mutant retained the ability to autopolysialylate, indicating that catalytic activity *per se* was not impacted by the replacements. The analogous residues in ST8SiaII, Arg^97^ and Lys^108^, were also found to be important for NCAM polysialylation, and their replacement did not impact polyST autopolysialylation (Fig. 5*B*) (110). In contrast, replacing selected ST8SiaIV PSTD basic residues (Arg^265^, Lys^276^, and Arg^277^) with alanine (R265A/K276A/R277A) eliminated its ability to polysialylate NCAM or autopolysialylate, suggesting these residues are involved in catalytic activity (chain initiation) and/or forming a basic surface that is essential for polySia chain elongation. In sum, selected PBR basic residues are essential for NCAM recognition, and selected PSTD residues may play a dual role in NCAM recognition as well as the processive elongation of polySia chains.

Indeed, later studies by Liao *et al.* (111) suggested direct interactions between the ST8SiaIV PSTD region and CMP–sialic acid donor as well as colominic acid (an α2,8-linked polymer of Sia residues used as a proxy for the growing polySia chain). Using NMR, they determined the secondary structure of a synthetic PSTD peptide and found that the PSTD is composed of an N-terminal short helix spanning from Lys^249^ to Val^251^ and a C-terminal long helix spanning from Ser^257^ to Arg^277^. They suggested that the short helix acts as a capture site for the CMP–Sia donor, allowing Sia to be easily transferred onto the growing polySia chain, whereas the long helix binds the polySia chain *via* electrostatic interactions. The presence of a significant number of hydrophobic residues in the long helix is also believed to guide polySia chain elongation *via* hydrophobic interactions. In contrast, the structural model of ST8SiaIV based on the X-ray crystal structure of ST8SiaIII obtained by Volkers *et al.* (112) suggests that the PSTD plays a role in glycan acceptor binding, as the C terminus of this region is proximal to the glycan acceptor binding sialyl-motif, SMS. This model effectively splits the PSTD into the acceptor-binding C-terminal region and the N-terminal region, which is spatially closer to PBR (Fig. 5*D*).

### Evidence for interaction between ST8SiaIV PBR and NCAM FN1 sequences

While the aforementioned results suggested that the basic residues in the PBR region, mainly Arg^82^ and Arg^93^ in ST8SiaIV and Arg^97^ and Lys^108^ in ST8SiaII, play a significant role in NCAM recognition, an interaction between these acidic residues in the NCAM FN1 domain and basic residues in the polyST PBR was first suggested by the competition experiments of Zapater *et al.* (113). They demonstrated that the ability of a catalytically inactive ST8SiaIV (H331K mutant) to compete with active ST8SiaIV to block NCAM polysialylation was eliminated by replacing Arg^82^ and Arg^93^ in the PBR with alanine. These results suggested that these PBR residues interacted with NCAM; however, a direct interaction between the acidic residues in NCAM FN1 and the basic residues in the polyST PBR was yet to be determined.

### Biophysical and structural evidence for a PBR–NCAM FN1 interaction

Efforts to obtain polyST proteins in the necessary quantities and with the required purity to enable their structural characterization were unsuccessful, and researchers had to rely on structural modeling and isolated domains that could be purified recombinantly. Bhide *et al.* (114) used isothermal titration calorimetry and NMR spectroscopy to evaluate the interaction of ST8SiaIV PBR and the NCAM FN1 domain. This approach demonstrated a dose-dependent 1:1 interaction between the NCAM FN1 domain and a His-SUMO-PBR peptide (residues 75–100) that could not be replicated by His-SUMO tag alone (114). When the residues of the FN1 acidic patch were replaced by arginine residues, or when Arg^82^ and Arg^93^ in the His-SUMO-PBR peptide were replaced by alanine residues, binding was lost, indicating the specificity of this interaction.

More detail about the PBR–FN1 interaction was obtained using NMR spectroscopy (114). Upon addition of the His-SUMO-PBR peptide to an ^15^N-labeled FN1 domain, residues showing dose-dependent perturbations included the FN1 acidic patch residues Asp^520^ and Glu^521^, in addition to Asp^498^ and Asp^506^ that are part of a larger acidic surface on the NCAM FN1 domain, as well as other previously characterized regions, including some residues in the Ig5–FN1 linker (Asp^498^, Thr^499^) and the GGVPI loop flanking this linker (Gly^526^ and Gly^527^) (Fig. 4). Notably, the side chain of Asp^498^ in the Ig5–FN1 linker is predicted to form a hydrogen bond with the main chain carbonyl of Gly^526^ (87) (Fig. 4, *inset*). Perturbations in NMR spectroscopy experiments suggest direct interactions between the partners; however, it is conceivable that the perturbations observed beyond the acidic patch were the result of relayed conformational change following the PBR–acidic patch interaction.

Further confirmation of these observations came from Zhou and Huang (115), who demonstrated an interaction of PBR residues, Arg^82^, Lys^83^, and Arg^93,^ with the NCAM FN1 domain using Wenxiang diagrams in conjunction with NMR studies. These diagrams elucidate the chain orientation of these PBR residues found in or near an α-helix spanning from Ser^75^ to Leu^89^ (Fig. 5*D*). Docking the NCAM Ig5–FN1 structure onto a model of ST8SiaIV allowed Volkers *et al.* (112) to predict that Glu^521^ in the NCAM FN1 acidic patch forms a salt bridge with Arg^93^ in the ST8SiaIV PBR and that basic residues in the PSTD region of ST8SiaIV interact with Glu^523^ in the NCAM FN1 acidic patch. Notably, no chemical shift perturbations associated with ST8SiaIV PBR binding by Glu^523^ were observed in the NMR study of Bhide *et al.* (114), even though mutating this residue significantly impacts NCAM polysialylation.

It is important to mention here that three-dimensional models based on the crystal structure of ST8SiaIII by the Strynadka and Zhou laboratories (111, 112, 115) suggested that the ST8SiaIV PBR and PSTD together form a positively charged groove on the surface of the enzymes (Fig. 5*D*). These and earlier results highlight the possibility that these regions may play multiple roles in the polysialylation process, including the interaction of the polySTs with protein substrates (recognition), with glycan acceptors (initiation), and/or with the growing polySia chain (elongation).

## The two-domain polysialylation paradigm: requirements for SynCAM 1 and NRP2 polysialylation

While NCAM remains the most abundant and well-characterized polyST substrate, the sequence requirements for two other glycoproteins, NRP2 and SynCAM1, are described below.

### NRP2 sequences required for ST8SiaIV recognition

**Figure 6:**
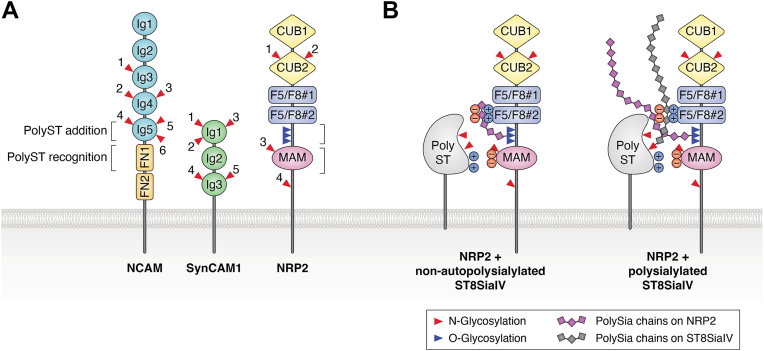
**Comparison of polyST recognition and polysialylation domains in NCAM, SynCAM1, and NRP2.***A*, the polySTs recognize sequences in NCAM FN1 that allow them to engage and polysialylate *N*-glycans on the adjacent Ig5 domain. Other data suggest that the polySTs must be able to engage with sequences in the Ig5 domain for productive polysialylation to occur. Like NCAM, both SynCAM1 and NRP2 have a recognition domain adjacent to the region modified by the glycans that are polysialylated. For SynCAM1, ST8SiaII recognizes sequences in the Ig2 domain, and this allows polysialylation of *N*-glycans in the adjacent Ig1 domain. For NRP2, the polySTs recognize sequences in the MAM domain that is adjacent to an extended region possessing *O*-glycans that are polysialylated. *B*, model for NRP2 polysialylation with emphasis on ST8SiaIV autopolysialylation. *Left panel* shows synthesis of shorter polySia chains by nonautopolysialylated ST8SiaIV, which remain tethered to NRP2 F5/F8#2 domain, blocking their polymerization. *Right panel* shows the elongation of polySia on NRP2 permitted because polySia chains on the ST8SiaIV bind the basic residues in the F5/F8#2 domain, thereby blocking the binding of the newly formed polySia chains on NRP2, allowing their further polymerization. PolySia chains on ST8SiaIV are shown as *gray diamonds*, and polySia chains on NRP2 are shown as *purple diamonds*. NCAM, neural cell adhesion molecule; NRP2, neuropilin 2; polyST, polysialyltransferase; SynCAM1, synaptic cell adhesion molecule 1.

NRP2 is a VEGF/class 3 semaphorin coreceptor with roles in axon guidance and vascularization (46). Like NCAM, NRP2 has a modular structure in its extracellular portion consisting of two complement C1r/C1s, Uegf, Bmp (CUB) domains, two coagulation factor 5/factor 8 (F5/F8) homology domains, and one meprin/a5 protein/μ tyrosine phosphatase (MAM) domain (Fig. 6, NRP2). Rollenhagen *et al.* (116) showed that NRP2 was exclusively polysialylated by ST8SiaIV on *O*-glycans in mature dendritic cells. Site-directed mutagenesis demonstrated that polySia was synthesized on *O*-glycans found in the linker region between the MAM domain and the second F5/F8 domain.

Using NRP2 domain deletion mutants, Bhide *et al.* (117) showed that the NRP2 MAM domain and the linker between the MAM domain and the second F5/F8 domain that contains the *O*-glycans that are polysialylated are sufficient for polysialylation. In addition, an NRP2 protein lacking the MAM domain neither showed polysialylation nor did it bind to ST8SiaIV in coimmunoprecipitation experiments.

To determine whether the MAM domain was necessary for NRP2 polysialylation, a chimeric protein approach was employed. As the related protein NRP1, which shares 44% sequence identity with NRP2 (116), was not reported to be polysialylated, the MAM domain of NRP2 was replaced with that of NRP1 (118). A significant decrease in polysialylation was observed for the resulting soluble chimeric protein. Unexpectedly, membrane-bound NRP1 was polysialylated at levels ∼50% of that observed for NRP2, and it was polysialylated to equivalent levels when its MAM domain was swapped with the MAM domain of NRP2.

Previously published studies explained this surprising observation. NRP1 undergoes continuous endocytosis and recycles to the cell surface even in the absence of any ligand (*e.g.*, VEGF) *via* the sequences in its cytoplasmic tail (119). It seemed possible that repeated exposure to overexpressed polySTs in Golgi and endosomal compartments may have resulted in NRP1 polysialylation despite suboptimal polyST recognition. A similar “reglycosylation” event has been previously reported by Duncan and Kornfeld (120) for the mannose-6-phosphate receptor. In support of this idea, a soluble, secreted NRP1-Fc lacking its transmembrane region and cytoplasmic tail was not polysialylated when coexpressed with ST8SiaIV, whereas a soluble, secreted NRP2-Fc was polysialylated (117). However, a soluble NRP2-Fc chimeric protein possessing the NRP1 MAM domain was still polysialylated, albeit at much lower levels, suggesting that the linker region may also contain secondary recognition elements, as previously observed for the NCAM Ig5 domain. Domain swap experiments involving the MAM domain as well as the linker region confirmed this notion (117). The NRP2 MAM domain also shared another element with the NCAM FN1 domain that was important for polysialylation—an acidic patch; mutating Glu^652^ and Glu^653^ in the NRP2 MAM domain to alanine eliminated NRP2 polysialylation (117).

### SynCAM1 sequences required for ST8SiaII recognition

SynCAM1 is a type 1 membrane protein containing three Ig domains in tandem (42) (Fig. 6, SynCAM1). Rollenhagen *et al.* (44) showed that *N*-glycans in the outermost Ig domain (Ig1) are polysialylated by ST8SiaII and that Ig2 must be present for polysialylation to occur. This indicated that the Ig2 domain likely carried recognition sites for ST8SiaII. Inspection of the Ig2 domain shows it contains several acidic residues that could form an acidic patch; however, the role of these in ST8SiaII recognition and polysialylation of SynCAM1 remains to be evaluated.

## PolyST autopolysialylation and its impact on the polysialylation of NCAM, SynCAM1, and NRP2

### Autopolysialylation of the polySTs

The polySTs, ST8SiaII and ST8SiaIV, are autopolysialylated. Muhlenhoff *et al.* (16) first found that recombinant ST8SiaIV was able to autocatalytically polysialylate its own *N*-glycans *in vitro*. Later work by the Colley laboratory demonstrated that both ST8SiaII and ST8SiaIV are autopolysialylated when expressed in COS-1 cells and that the enzymes were the only polysialylated proteins in these cells (17, 75, 121). In addition, a number of investigators identified the *N*-glycans in each polyST that carry polySia (see Fig. 5 for sites of ST8SiaIV autopolysialylation) (16, 17, 75, 79). The majority of polySia is found on the glycan modifying Asn^74^ in ST8SiaIV, whereas the polySia on ST8SiaII is more equally distributed on the three glycans modifying Asn^89^, Asn^219^, and Asn^234^ (75, 79). Work by Bhide *et al.* (118) indicated that a single polyST molecule can autopolysialylate its own glycans rather than requiring a partner molecule to crosspolysialylate its *N*-glycans.

### Role of polyST autopolysialylation in NCAM, NRP2, and SynCAM1 polysialylation

Autopolysialylation of the polySTs is differentially required for substrate polysialylation. Work by Close *et al.* (75, 121) demonstrated that ST8SiaII and ST8SiaIV autopolysialylation was not required for, but did enhance, NCAM polysialylation, resulting in higher molecular mass polysialylated NCAM molecules. In contrast, the work of Muhlenhoff *et al.* (16, 122) suggested that ST8SiaII autopolysialylation was required for NCAM polysialylation based on their observation that replacing all three Asn glycosylation sites that carried the *N*-glycans that were autopolysialylated with glutamine created an enzyme unable to polysialylate NCAM. However, Close *et al.* (121) showed that while the triple glutamine replacement mutant was indeed unable to polysialylate NCAM, an ST8SiaII triple serine mutant did polysialylate NCAM, although at lower levels than the unaltered protein. In a similar vein, Angata *et al.* (123) demonstrated that nonautopolysialylated ST8SiaIV expressed in insect cells, which inherently lack sialylation machinery, was able to polysialylate NCAM.

As with NCAM, ST8SiaII autopolysialylation was not essential for the polysialylation of SynCAM1, although the polysialylated SynCAM1 proteins migrated with a lower molecular mass, suggesting that the polySia chains synthesized by the nonautopolysialylated enzyme were either fewer or shorter. In contrast, NRP2 was not polysialylated by either of two nonautopolysialylated but catalytically active ST8SiaIV mutants (118). It is important to mention here that the ability of the nonautopolysialylated polySTs to polysialylate NCAM and SynCAM1 indicates that the polySia chain is not preassembled on the polySTs and then transferred *en bloc* to these substrates’ glycans. Why polyST autopolysialylation enhances NCAM and SynCAM1 polysialylation and is required for NRP2 polysialylation is not clear, but some possibilities are provided in the *Discussion* section.

## Comparison of polyST PBR sequences required for polyST autopolysialylation, NCAM, NRP2, and SynCAM1 polysialylation

The probable steps in the polysialylation of a glycoprotein include a recognition event that would involve protein–protein interaction, followed by an initiation event (the addition of the initial α2,8-sialic acid residue), and then a polySia chain elongation event potentially facilitated by an extended polybasic groove on the polyST surface. In the assay systems used in the aforementioned studies, it would be difficult to distinguish initiation from elongation, as the antibodies used to detect polysialylation recognized chains of five to eight units of sialic acid or more. Based on the experiments that suggest polyST autopolysialylation is a “self” polysialylation (118), the recognition event would not apply in this case. Consequently, any polyST mutants (PBR mutants) that decrease autopolysialylation would be expected to impact initiation and/or elongation of polySia chains, and this could include not just an impact on catalytic activity *per se* but potentially an impact on accessibility of the enzyme’s *N*-glycans for autopolysialylation. In the former case, where a polyST mutant blocked autopolysialylation because of an impact on initiation or chain elongation, those mutants should have a negative impact on glycoprotein substrate polysialylation too. Results described below are summarized in Figure 5*B*.

For the PBR mutants of ST8SiaIV, the K99A mutation substantially decreases both autopolysialylation and NRP2 polysialylation, with no impact on NRP2 recognition or NCAM recognition or polysialylation. The absence of an impact on NCAM polysialylation is significant here and implies that the K99A mutant does not alter catalytic activity *per se* but instead is potentially compromising ST8SiaIV autopolysialylation from a more structural/accessibility standpoint, and then this in turn blocks NRP2 polysialylation, which requires ST8SiaIV autopolysialylation. Results for the ST8SiaIV R93A mutant are more straightforward, leading to the conclusion that this residue is essential for NCAM recognition and polysialylation and does not play a role in either ST8SiaIV autopolysialylation or NRP2 recognition and polysialylation. On the other hand, the ST8SiaIV R82A mutant has a moderate impact on autopolysialylation (decreases ∼50%) and decreases NCAM recognition and polysialylation to similar extents but has a much more significant negative impact on NRP2 recognition and polysialylation. Taken together, these data suggest that Arg^82^ may play a role in both NCAM and NRP2 recognition, and the more severe negative impact on NRP2 recognition and polysialylation could be based on the role the enzyme autopolysialylation plays in this substrate’s recognition and/or polysialylation.

The situation with the PBR residues in ST8SiaII seems more complicated. Mutation of nearly every residue in the ST8SiaII PBR seems to impact NCAM recognition, but only the R97A, K108A, K114A, and K118A mutants negatively impact NCAM polysialylation. In contrast, the K102A, K114A, and K118A mutants most severely impact SynCAM1 polysialylation, with Lys^102^ and Lys^114^ making the major contributions to recognition. Notably, autopolysialylation is severely inhibited for the K114A and K118A mutants. The negative impact these mutants have on autopolysialylation and NCAM and SynCAM1 polysialylation makes it tempting to conclude that these mutants have an impact on polySia chain initiation and elongation. However, these residues also play a significant role in NCAM recognition, suggesting a dual purpose, at least for NCAM.

In sum, NCAM is primarily recognized by analogous PBR residues in ST8SiaIV (Arg^82^ and Arg^93^) and ST8SiaII (Arg^97^ and Lys^108^), with some contributions from other residues. For NRP2, ST8SiaIV Arg^82^ may play a major role in recognition, with some lesser contributions from Lys^83^ and Arg^97^. SynCAM1 recognition by ST8SiaII seems to be mainly mediated by Lys^102^ with possible lesser contributions by Arg^87^ and Lys^114^. The contributions of Lys^114^ and Lys^118^ in ST8SiaII to substrate recognition and polysialylation are complicated by the fact that alanine replacements of these residues severely compromise enzyme autopolysialylation. Returning to ST8SiaIV autopolysialylation, Lys^99^ is required for autopolysiaylation and NRP2 polysialylation (but not recognition), likely because this substrate’s polysialylation requires ST8SiaIV autopolysialylation.

## Discussion

The studies summarized above demonstrate that mammalian polysialylation is a protein-specific process that requires the polySTs, ST8SiaII and ST8SiaIV, to recognize and bind amino acid residues in a region of the glycoprotein substrate adjacent to the region carrying the glycans that are polysialylated (Fig. 6*A*). For NCAM and NRP2, specific acidic residues have been identified that mediate the interaction with the polySTs (114, 117). Basic residues in the PBR of the polySTs have been identified that interact with the acidic residues in the substrates (110, 113, 118). A direct interaction between basic amino acids in the ST8SiaIV PBR and acidic amino acids in the NCAM FN1 domain has been demonstrated using biophysical methods (114). In addition, for both NCAM and NRP2, data suggest that there are secondary interactions in the modified domain that contribute to recognition and polysialylation by the polySTs (92, 117).

The studies described in this review suggest a two-domain paradigm, where the polySTs recognize sequences in one domain/region of a glycoprotein substrate and modify glycans in an adjacent domain/region (Fig. 6*A*). These interactions are not only somewhat constrained but also enhanced by the membrane association of both the substrate and the polyST. Soluble forms of the substrates are polysialylated to a lesser extent than their membrane-associated counterparts (79). In addition, changing the distance of the substrate recognition site from the membrane appears to weaken the polyST–substrate interaction. For example, alanine replacement of FN1 acidic patch residues eliminates the polysialylation of membrane-associated NCAM Ig5–FN1 but not membrane-associated Ig5–FN1–FN2 or full-length NCAM, whereas more dramatic arginine replacements of these residues impact the polysialylation of all three proteins (83).

Residues in two regions unique to the polySTs, PBR and PSTD, may play multiple roles in substrate recognition and polySia chain elongation. Work described in this review highlighted the role of specific basic residues in the polyST PBR for NCAM, SynCAM1, and NRP2 recognition and binding. Other studies suggested that mammalian and bacterial polySTs, as processive enzymes, may have extended acceptor substrate interaction sites that allow them to continue to engage their substrates as the polySia chain is polymerized. For example, evaluation of the X-ray crystal structure of the polyST from *Mannheimia haemolytica* serotype A2 identified a positively charged groove between the N-terminal and C-terminal Rossman domains that form the GT-B fold that Lizak *et al.* (124) predicted may bind the growing polySia chain. In support of this prediction in the mammalian system, the work of Nakata *et al.* (109) supported the possibility that basic residues in the ST8SiaIV PSTD may serve as a positively charged surface that stabilizes the growing polySia chain. This was followed by a later study by Peng *et al.* (125) that demonstrated that heparin, a negatively charged glycopolymer that directly binds the PSTD, can inhibit NCAM polysialylation *in vitro.*

Structural studies supported these roles for the conserved polyST PBR and PSTD sequences. Volkers *et al.* (112) solved the X-ray crystal structure of ST8SiaIII, allowing them to model the structure of ST8SiaIV and dock the NCAM Ig5–FN1 structure on that modeled structure. What they found verified and extended previous studies by suggesting that the PBR interacts with the NCAM FN1 acidic patch (salt bridge between NCAM FN1 Glu^521^ and ST8SiaIV PBR Arg^93^) and that the PSTD basic residues interact with Glu^523^ in the NCAM FN1 acidic patch. Another model of ST8SiaIV based on the structure of ST8SiaIII (Fig. 5*D*) also highlights the close proximity of the PBR and the PSTD to one another and to the sialyl motifs involved in glycan substrate recognition and catalytic activity and suggests that basic residues in this extended region may serve as an electropositive groove that promotes continued engagement with the growing polySia chain. Taken together, it is likely that the polyST PBR and PSTD regions interact with the substrate protein as well as the growing polySia chain.

The autopolysialylation of the polySTs differentially impacts substrate polysialylation. For NCAM and SynCAM1 *N*-glycan polysialylation, polyST autopolysialylation is not required for, but does enhance, these substrates’ polysialylation. In contrast, for NRP2, ST8SiaIV autopolysialylation is required for the polysialylation of its O-linked glycans. One possibility is that the polySia on the polySTs may block the growing polySia chain from interacting with basic sequences on the substrates that would inhibit the elongation of the growing chain along the polyST basic groove, likely formed by residues in the PBR and PSTD. This might be accentuated for the NRP2 *O*-glycans that are shorter than *N*-glycans, effectively blocking chain extension (Fig. 6*B*). With this possibility in mind, a comparison of the relative charge profile of the NRP2 F5/F8 #2 domain and the NCAM Ig4 domain, both adjacent to the region carrying the polysialylated glycans in these proteins, revealed that the former is significantly more basic (pI ∼ 8.3) compared with the latter (pI ∼ 5.2). Based on this information, one could envision that ST8SiaIV polySia chains may electrostatically block the basic F5/F8 #2 domain from interacting with the NRP2 polySia chains, allowing the elongation of these chains along the ST8SiaIV basic groove.

Many mechanistic and functional questions still remain. For example, will the two-domain paradigm hold up for other substrates? What is the mechanism of polySia chain termination, and what controls polySia chain length? Is there a substrate hierarchy; is NCAM the most highly expressed polysialylated protein, or is it the best polyST substrate? When more than one polysialylated protein is expressed in the same cell type at the same time, what role is polySia playing (*e.g.*, NRP2 and CCR7 in mature dendritic cells, NRP2 and ESL-1 in THP macrophages and glial cells)?. Are the modified proteins simply carriers for polySia? Or are protein activities modified by polySia? Or both?

As a post-translational modification, polySia plays both beneficial and detrimental roles in mammals, prompting scientists to find ways to block and promote its biosynthesis. The dual role of polySia as a repulsive force negatively modulating adhesive interactions between proteins and cells and as an attractive agent binding a variety of biological molecules allows it to both block cell interaction and modulate signaling pathways, thereby impacting nervous system development, immune cell development and function, cancer cell survival and metastasis, and tissue regeneration (2, 4, 21, 126, 127, 128).

Polysialylated NCAM is highly expressed in many types of cancers, including neuroblastoma, lung cancer, breast cancer, and pancreatic cancer, and its presence is associated with late stage, poorly differentiated, and highly invasive and metastatic disease (2, 23). Early work by Scheidegger *et al.* (129), in which they compared NIH H69 small cell lung carcinoma lines expressing varying amounts of polySia, demonstrated that polySia-positive cells formed more colonies in soft agar and formed more intracutaneous metastases when injected into nude mice. More recently, Elkashef *et al.* (28) demonstrated that the presence of polySia allows continued survival and migration of glioma and neuroblastoma cells under hypoxic conditions similar to those that are found in tumors. Undoubtedly, it would be beneficial to find ways to inhibit the polySTs in cancer cells, and particularly, ST8SiaII, which is more highly expressed in tumors (23). To this end, Al-Saraireh *et al.* (130) used CMP as a competitive inhibitor of ST8SiaII to block NCAM polysialylation and demonstrated decreased migration of treated cancer cells. Likewise, many previous studies (131, 132, 133, 134, 135, 136, 137) have identified sialic acid analogs (*e.g.*, 3F-Neu5Ac) and CMP analogs (*e.g.*, 2′-*O*-methyl CMP) as inhibitors of sialyltransferases and polySTs. However, these inhibitors have the potential to be nonspecific and to inhibit multiple sialylation reactions and consequently could be less useful. Could we block polyST substrate recognition by using the information we have obtained about the glycoprotein substrate and polyST sequences involved in this process? An acidic patch mimetic might work *in vitro*, but in the cell, polySTs modify their substrates in the Golgi compartment, complicating the delivery of the competitive inhibitor. Novel therapeutics that leverage gene therapy, such as siRNA/antisense oligonucleotides, can also be explored to reduce the expression of polySTs in disease.

Therapeutic proteins, either chemically conjugated to bacterial polySia or directly modified by bacterial polySTs, have been shown to exhibit increased circulating half-lives (138, 139). A complication of modifying therapeutic proteins using bacterial polySTs is that there is no site (or protein) specificity, leaving open the possibility that the added polySia chains may inhibit protein activity. It seems plausible that an acidic recognition site could be engineered into a therapeutic protein with a known three-dimensional structure, allowing it to be modified more specifically by a mammalian polyST. In another scenario, injection of a bacterial polyST into a mouse brain has been shown to lead to the polysialylation of multiple proteins and could be used to promote nervous system repair (140). With this example in mind, could the coinjection–coexpression of an optimal substrate (NCAM) and a polyST achieve the same or a better effect? While there is much work to do to understand how other substrates are recognized, how the polySia chain is polymerized, and what signals its final release, we hope that the mechanistic understanding captured in this review can be leveraged for the development of more effective therapeutics targeting polysialylation machinery.

## Conflict of interest

The authors declare that they have no conflicts of interest with the contents of this article.
